# Associations of CKD risk factors and longitudinal changes in urine biomarkers of kidney tubules among women living with HIV

**DOI:** 10.1186/s12882-021-02508-6

**Published:** 2021-08-30

**Authors:** Anthony N. Muiru, Rebecca Scherzer, Simon B. Ascher, Vasantha Jotwani, Carl Grunfeld, Judy Shigenaga, Kimberly A. Spaulding, Derek K. Ng, Deborah Gustafson, Amanda B. Spence, Anjali Sharma, Mardge H. Cohen, Chirag R. Parikh, Joachim H. Ix, Michelle M. Estrella, Michael G. Shlipak

**Affiliations:** 1 Kidney Health Research Collaborative, Department of Medicine, San Francisco Veterans Affairs Health Care System and University of California, San Francisco, CA USA; 2 Department of Medicine, Division of Nephrology, University of California, 533 Parnassus Avenue, U404, Box 0532, San Francisco, CA 94143 USA; 3 Division of Hospital Medicine, University of California Davis, Sacramento, CA USA; 4 Department of Epidemiology, Johns Hopkins Bloomberg School of Public Health, Baltimore, MD USA; 5 Department of Neurology, The State University of New York Downstate Health Sciences University, Brooklyn, New York, USA; 6 Division of Infectious Diseases, Department of Medicine, Georgetown University Medical Center, Washington, DC, USA; 7 Department of Medicine, Albert Einstein College of Medicine, Bronx, New York, USA; 8 Department of Medicine, Stroger Hospital and Rush University, Chicago, IL USA; 9 Division of Nephrology, Department of Medicine, Johns Hopkins School of Medicine, Baltimore, MD USA; 10 Division of Nephrology-Hypertension, University of California, San Diego, CA USA

**Keywords:** Urine biomarkers HIV CKD

## Abstract

**Background:**

Novel urine biomarkers have enabled the characterization of kidney tubular dysfunction and injury among persons living with HIV, a population at an increased risk of kidney disease. Even though several urine biomarkers predict progressive kidney function decline, antiretroviral toxicity, and mortality in the setting of HIV infection, the relationships among the risk factors for chronic kidney disease (CKD) and urine biomarkers are unclear.

**Methods:**

We assessed traditional and infection-related CKD risk factors and measured 14 urine biomarkers at baseline and at follow-up among women living with HIV in the Women’s Interagency Health Study (WIHS). We then used simultaneously adjusted multivariable linear regression models to evaluate the associations of CKD risk factors with longitudinal changes in biomarker levels.

**Results:**

Of the 647 women living with HIV in this analysis, the majority (67%) were Black, the median age was 45 years and median follow-up time was 2.5 years. Each traditional and infection-related CKD risk factor was associated with a unique set of changes in urine biomarkers. For example, baseline hemoglobin a1c was associated with worse tubular injury (higher interleukin [IL]-18), proximal tubular reabsorptive dysfunction (higher α1-microglobulin), tubular reserve (lower uromodulin) and immune response to injury (higher chitinase-3-like protein-1 [YKL-40]). Furthermore, increasing hemoglobin a1c at follow-up was associated with further worsening of tubular injury (higher kidney injury molecule-1 [KIM-1] and IL-18), as well as higher YKL-40. HCV co-infection was associated with worsening proximal tubular reabsorptive dysfunction (higher β2-microglobulin [β2m]), and higher YKL-40, whereas HIV viremia was associated with worsening markers of tubular and glomerular injury (higher KIM-1 and albuminuria, respectively).

**Conclusions:**

CKD risk factors are associated with unique patterns of biomarker changes among women living with HIV, suggesting that serial measurements of multiple biomarkers may help in detecting and monitoring kidney disease in this setting.

**Supplementary Information:**

The online version contains supplementary material available at 10.1186/s12882-021-02508-6.

## Introduction

Standard markers of kidney disease, including estimated glomerular filtration rate (eGFR) and albuminuria, predominantly reflect glomerular dysfunction and injury and do not adequately detect kidney tubular health [1–3]. The tubules are the site of key physiologic functions including reabsorption and secretion of solutes and fluids to maintain homeostasis, as well as hormone production and numerous metabolic activities [4–9]. Urine tubule biomarkers have enabled the characterization of tubular reabsorption and synthetic functions, injury, reserve, and fibrosis, allowing a more comprehensive assessment of the nephron [10, 11]. In addition, several urine biomarkers are associated with chronic kidney disease (CKD) onset, rapid declines in kidney function, medication toxicity and mortality risk, independent of eGFR and albuminuria [12–21]. However, the relationship between various CKD risk factors and longitudinal changes in urine biomarker levels is unclear.

CKD risk factors in the setting of HIV infection often appear in combination and include traditional risk factors, such as diabetes and hypertension [22], as well as infection-related risk factors, such as uncontrolled viremia [23], hepatitis C virus (HCV) co-infection [24], and exposure to potentially nephrotoxic antiretroviral therapy (ART) [25]. These risk factors may simultaneously cause injury at diverse parts of the nephron. A critical question for clinical practice is whether we can utilize changes in urine biomarker levels to discern which risk factors contribute the most to kidney disease in an individual patient, and thus inform treatment decisions.

Ultimately, we envision using this information to develop parsimonious algorithms that integrate longitudinal changes in biomarker levels with clinical data to better detect and manage kidney disease. However, most studies to date have predominantly examined the association of CKD risk factors with biomarkers cross-sectionally [26–30]. To advance the kidney biomarker research field forward we need to ascertain whether these risk factors contribute to changes in the biomarkers over time. In this study, we evaluated the association of traditional and infection-related CKD risk factors with longitudinal changes in urinary biomarkers among women with HIV in the Women’s Interagency HIV Study (WIHS).

## Methods

### Study population and study design

WIHS is an ongoing, longitudinal prospective cohort of women who are either infected with HIV or considered at high-risk for acquiring HIV [31, 32]. Briefly, WIHS initially enrolled a total of 4909 women in 1994–1995 and 2001–2002 from various study sites in the United States. Standardized questionnaires to obtain sociodemographic and clinical information are administered during semi-annual study visits, as well as physical examinations and collection of biological specimens. Beginning in 2009, urine samples were collected and stored annually.

In this nested study within WIHS, we included 647 women living with HIV who had two available serial urine and serum specimens and with preserved kidney function (eGFR ≥60 ml/min/1.73m^2^) at the time of the first specimen collection. The first urine specimen was collected between October 2009 and March 2011, and the second urine specimen was collected a median of 2.5 years later. The Institutional Review Board (IRB) at the University of California San Francisco (UCSF) approved the study protocol, and informed consent was obtained from all study participants. All methods were carried out in accordance with relevant guidelines and regulations.

### Measurement of urine biomarkers of kidney health

Clean catch spot urine specimens were collected at study visits, refrigerated immediately after collection, and subsequently centrifuged. Supernatants were then stored in 1-mL aliquots at − 80 °C until biomarker measurement was undertaken, without prior freeze-thaw. We measured 14 urine biomarkers that represent various aspects of kidney health including: 1) glomerular/ endothelial injury: urine albumin (Ualb) and osteopontin (OPN); 2) proximal tubular reabsorptive dysfunction: α1-microglobulin (α1m), β2-microglobulin (β2m), cystatin C (CysC), and trefoil factor 3 (TFF3); 3) tubular injury: kidney injury molecule-1 (KIM-1), interleukin (IL)-18, clusterin, and neutrophil gelatinase-associated lipocalin (NGAL); 4) tubular reserve: uromodulin (UMOD) and epidermal growth factor (EGF); 5) tubulointerstitial injury and fibrosis: monocyte chemoattractant protein-1 (MCP-1); and 6) immune response to injury: chitinase-3-like protein-1 (YKL-40) [10, 33–35]. Several of these biomarkers can be classified into multiple categories, and the current categorization is simplistic in order to facilitate better communication of our results. All urine biomarkers were measured in duplicates. Urine creatinine was measured using a colorimetric assay (intra-assay coefficient of variation [CV] 3.3%, RnD Systems, Minneapolis, MN) and α1m was measured using a commercial assay (CV 4.1%, Siemens BN II Nephelometer, Munich, Germany). All other urine biomarkers were measured using multiplex immunoassays from Meso Scale Discovery (MSD, Rockville, MD). The MSD platform utilizes a proprietary multi-array technology with electrochemiluminescence detection of biomarkers, and included Kidney Injury Panel 3 which measured clusterin (CV 8.8%), KIM-1 (CV 4.7%), and TFF-3 (CV 5.3%). Kidney Injury Panel 5 measured urine albumin (CV 5.7%), β2m (CV 4.9%), EGF (CV 7.2%), and UMOD (CV 4.0%). A custom MSD panel was used to measure IL-18 (CV 4.7%) and YKL-40 (CV 2.5%).

### Definitions of CKD risk factors

We categorized candidate CKD risk factors as either traditional or infection-related. Traditional risk factors included: 1) age; 2) race (self-reported Black or other vs. White race); 3) diabetes mellitus; 4) hemoglobin a1c; 5) history of hypertension; 6) systolic and diastolic blood pressure; 7) low-density lipoprotein (LDL) and high-density lipoprotein (HDL); 8) statin use; 9) body mass index (BMI); and 10) current or past smoking. Infection-related risk factors included: 1) HCV co-infection; 2) plasma HIV-1 RNA (current and peak viral load); 3) CD4+ cell count (current and nadir); 4) serum albumin; 5) any ART use; 6) tenofovir disoproxil fumarate (TDF) use; 7) ritonavir use; 8) history of AIDS; and 9) duration of HIV infection.

Consistent with national guideline definitions and with prior WIHS analyses, diabetes mellitus was defined as: hemoglobin A1c ≥6.5%, fasting plasma glucose ≥126 mg/dL (7 mmol/L) or self-reported history of diabetes on anti-diabetic medications [36]. Hypertension was defined as: two consecutive measurements of systolic blood pressure (SBP) ≥140 mmHg, or diastolic blood pressure (DBP) ≥90 mmHg, or self-reported history of hypertension on antihypertensive medications [37]. HCV co-infection was defined as having a positive HCV antibody result confirmed with detectable HCV RNA. Plasma HIV RNA viral load was measured using the Roche COBAS AmpliPrep/COBAS TaqMan HIV-1 Test (LLD of 20 or 48 copies HIV RNA/mL). Serum creatinine-based eGFR was calculated using the CKD-EPI equation [38].

### Statistical analysis

We summarized demographic and clinical characteristics at the baseline and follow-up biomarker collection visits (baseline in 2009–2011 and follow-up [median of 2.5 years later]). We considered baseline CKD risk factors and changes in these risk factors between biomarker visits as exposures of interest. Our main outcome of interest was changes in biomarker levels over time. We first evaluated associations of CKD risk factors with biomarker levels using separate unadjusted linear regression models with robust Huber-White standard errors. We then modeled all the CKD risk factors (including both baseline and changes in risk factors) and all the biomarkers in combination using the multivariable sparse group least absolute shrinkage and selection operator (MSG-LASSO) method for variable selection [39]. This method is appropriate for settings involving both multiple predictors and multiple outcomes, and is able to produce a sparse solution, removing less influential variables and groups. Finally, we used multivariate simultaneous linear equations (constructed with three-stage least squares), retaining only variables selected by MSG-LASSO, to account for correlations between urine biomarkers and to create confidence intervals around the coefficient for each risk factor/biomarker dyad. This method is more appropriate than individual regression models given the inter-relatedness of the biomarkers.

In all models, biomarker concentrations were log-transformed to normalize their distributions and standardized to the same scale (mean 0, SD 1), and results were reported as standardized beta coefficients with 95% confidence intervals (CIs) to facilitate comparisons between each CKD risk factor and urine biomarker associations. For example, an estimate of 0.2 indicates a 1 standard deviation (SD) increase in a CKD risk factor is associated with a 0.2 SD increase in a biomarker level. Finally, we controlled for urine creatinine as a separate covariate, rather than indexing as urine biomarker/creatinine ratios, in all analyses to account for urine tonicity. Although our multivariate models mutually adjusted for multiple CKD risk factors and analyzed all 14 biomarkers in combination, we have summarized 7 key CKD risk factors and 8 biomarkers below which were selected based on clinical utility and prior literature showing strong associations with kidney disease. The 7 CKD risk factors included hemoglobin a1c, systolic blood pressure, serum albumin, HCV, CD4 count, HIV viral load and TDF duration, while the 8 biomarkers included Ualb, α1m, β2m, KIM-1, IL-18, UMOD, EGF and YKL-40. Full results including all risk factors, covariates and biomarkers analyzed can be found in Supplemental Table 1.

Penalized regression was performed using the R package *MSGLasso*. All other analyses were performed using the SAS system, version 9.4 (SAS Institute, Inc., Cary, NC).

## Results

Demographic and CKD risk factors at baseline and follow-up are presented in Table 1. Of the 647 women living with HIV in this analysis, the majority (67%) self-identified as Black, and the median age at baseline was 45 years (IQR: 40, 51). Median time to second biomarker measurement was 2.5 years (IQR 2.4–2.5 years). ART usage was 75% at baseline and 85% at follow-up; other markers of HIV control such as median CD4 count and HIV viral load improved over time. However, the proportion of participants with non-infectious comorbidities including diabetes and hypertension increased in prevalence during follow-up. Nearly all participants (97%) had normal or mildly decreased kidney function by eGFR (> 60 ml/min) at both baseline and follow-up.

**Table 1:** Summary of baseline and follow-up demographic and clinical characteristics of women living with HIV included in this study

Parameter	Baseline ***n*** = 647	Follow-up ***n =*** 647
Calendar year, mean ± SD	2009 ± 0.5	2012 ± 0.3
Race and/or ethnicity, N (%)		
African American	432 (67%)	
Other	100 (15%)	
White	115 (18%)	
Hispanic	133 (21%)	
Age, years, median (IQR)	45 (40, 51)	48 (43, 53)
Smoking, N (%)		
Current	249 (38)	229 (35)
Past	210 (32)	232 (36)
Never	188 (29)	186 (29)
Diabetic, N (%)	130 (20)	147 (23)
Hemoglobin A1c, %, median (IQR)	5.6 (5.3, 5.9)	5.7 (5.4, 5.9)
Systolic Blood Pressure, mmHg, median (IQR)	117 (108, 131)	118 (107, 132)
Diastolic Blood Pressure, mmHg, median (IQR)	73 (67, 81)	73 (67, 81)
Hypertension, N (%)	229 (35)	267 (41)
Antihypertensive use, N (%)	171 (26)	213 (33)
LDL, mg/dL, median (IQR)	93 (76, 118)	97 (74, 117)
HDL, mg/dL, median (IQR)	51 (40, 61)	51 (41, 65)
TG, mg/dL, median (IQR)	103 (75, 147)	107 (76, 152)
Statin use, N (%)	93 (14)	109 (17)
History of CVD, N (%)	5 (1)	5 (1)
BMI (kg/m^2^), median (IQR)	29 (25, 34)	29 (25, 34)
Waist Circumference (cm), median (IQR)	94 (86, 107)	95 (85, 108)
Duration of HIV infection (y), median (IQR)	14 (8, 15)	17 (10, 17)
Current ART, N (%)		
Any ART use	487 (75)	548 (85)
NRTI use	483 (75)	537 (83)
NNRTI use	202 (31)	221 (34)
PI use	274 (42)	302 (47)
TDF use	396 (61)	451 (70)
Current CD4, cells/μL, median (IQR)	518 (343, 730)	537 (365, 756)
Nadir CD4, cells/μL, median (IQR)	213 (113, 307)	200 (98, 290)
History of AIDS, N (%)	233 (36)	250 (39)
Plasma HIV RNA < 80 copies/mL, N (%)	386 (60)	447 (69)
Peak HIV RNA > 10 K copies/mL, N (%)	510 (79)	521 (81)
Hepatitis C, N (%)	124 (19)	130 (20)
Heroin use, N (%)	8 (1)	9 (1)
eGFR mL/min/1.73m^2^, median (IQR)	104 (89, 117)	100 (83, 115)
eGFR < 60 mL/min/1.73m^2^, N (%)	0	17 (2.6%)

*LDL* low-density lipoprotein (LDL), *HDL* high-density lipoprotein, *TG* triglycerides, *BMI* Body mass index, *ART* Antiretroviral therapy, *NRTI* Nucleoside reverse transcriptase inhibitors, *NNRTI* Non-nucleoside reverse transcriptase inhibitors, *PI* Protease inhibitor, *TDF* Tenofovir Disoproxil Fumarate, *eGFR* estimated glomerular filtration rate

In our fully adjusted models, we observed that each traditional CKD risk factor was associated with a distinct set of urine biomarker changes (Table 2). For example, baseline hemoglobin a1c was associated with several dimensions of worsening kidney health including proximal tubular reabsorptive dysfunction (higher α1m), tubular injury (higher IL-18), tubular reserve (lower UMOD) and immune response to injury (higher YKL-40). Additionally, changes in hemoglobin a1c during follow-up were simultaneously associated with worsening tubular injury (higher KIM-1 and IL-18) and immune response to injury (higher YKL-40). In contrast, baseline systolic blood pressure was associated with worse markers of glomerular injury (higher Ualb) and tubular reserve (lower EGF).

**Table 2:**
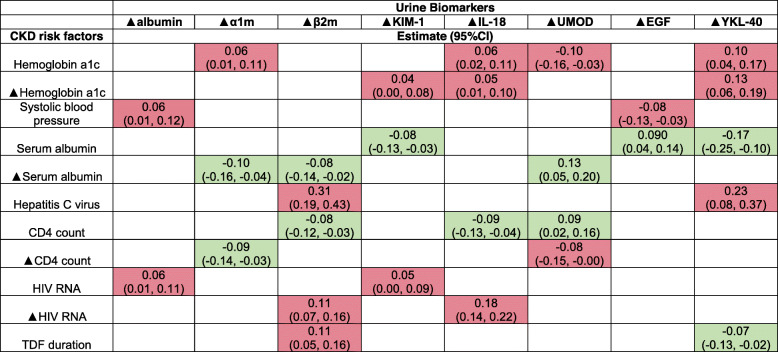
Simultaneous multivariable adjusted associations of baseline and follow-up CKD risk factors with longitudinal changes in urine biomarker levels among HIV-positive women

We modeled biomarkers in combination using the multivariable sparse group least absolute shrinkage and selection operator (MSG-LASSO) method for variable selection. Red shading indicates worsening marker of kidney markers, while green shading indicates improving kidney biomarkers. Blank boxes indicate variables that were not selected by the MSG-LASSO method. We have summarized 7 key CKD risk factors and 8 biomarkers which were selected based on clinical utility and prior literature showing strong associations with kidney disease. Estimates are reported as standardized regression coefficients (e.g 1 standard deviation (SD) increase in hemoglobin a1c is associated with a 0.06 SD increase in α1m). **▲:** change in risk factor or biomarker level; α1m: α1-microglobulin; β2m: β2-microglobulin; KIM-1: kidney injury marker-1; IL-18: interleukin 18; UMOD: uromodulin; EGF: epidermal growth factor; YKL-40: chitinase-3-like protein-1; TDF: Tenofovir Disoproxil Fumarate.

Similar to traditional CKD risk factors, each infection-related risk factor was associated with unique patterns of urine biomarker changes (Table 2). For example, baseline HCV co-infection was associated with worsening reabsorptive dysfunction (higher β2m) and immune response to injury (higher YKL-40), while baseline HIV viral load was associated with tubular (higher KIM-1) and glomerular (higher Ualb) injuries. Increasing HIV viral load during follow-up was additionally associated with worsening reabsorptive dysfunction (higher β2m) and tubular injury (higher IL-18). Longer TDF duration was associated with worsening reabsorptive dysfunction (higher β2m), but with improving markers of immune response to injury (lower YKL-40). In contrast, higher serum albumin concentrations at baseline were associated with improvement in tubular reabsorptive dysfunction, immune response and tubular reserve (lower KIM-1, YKL-40, and higher EGF, respectively), as were increasing serum albumin concentrations over time (lower α1m, β2m, and higher UMOD). Higher CD4 count at baseline was associated with improvements in reabsorptive dysfunction, tubular injury, and reserve (lower β2m and IL-18 and higher UMOD, respectively) and increasing CD4 count at follow-up was associated with improved reabsorptive dysfunction (decreasing α1m and β2m). Full results including all risk factors, and all biomarkers analyzed can be found in Supplemental Table 1.

**Fig. 1:**
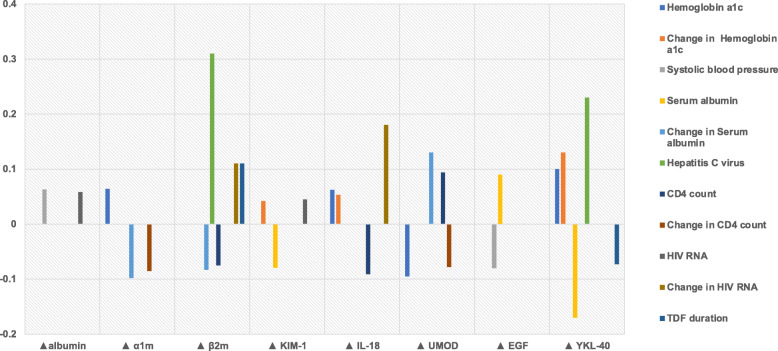
The vertical axis shows standardized regression coefficients, horizonatal axis shows changes in each urinary biomarker. α1m: α1-microglobulin; β2m: β2-microglobulin; KIM-1: kidney injury marker-1; IL-18: interleukin 18; UMOD: uromodulin; EGF: epidermal growth factor; YKL-40: chitinase-3-like protein-1; TDF: Tenofovir Disoproxil Fumarate

We then plotted the results shown in Table 2 in order to better identify the set of CKD risk factors that are associated with each individual urine biomarker. We noted that each urine biomarker changes were influenced by multiple traditional and infection-related CKD risk factors, but their associations varied in magnitude (Fig. 1). For example, baseline hemoglobin a1c, change in hemoglobin a1c, baseline CD4 count and change in HIV RNA viral load were all associated with varying levels of changes in IL-18, a marker of tubular injury (standardized beta coefficients of + 0.06, + 0.05, − 0.09, and + 0.18, respectively). In contrast, baseline hemoglobin a1c, changes in serum albumin and CD4 count were associated with different levels of α1m, a marker of proximal tubular reabsorptive dysfunction (standardized beta coefficient + 0.06, − 0.10 and − 0.09, respectively).

## Discussion

In this study, we evaluated the associations of multiple CKD risk factors with longitudinal changes in a panel of 14 urine biomarker levels in a contemporary cohort of women living with HIV. We observed that each traditional or infection-related CKD risk factor was associated with a distinct pattern of changes in biomarker levels. These findings suggest that the impact of CKD risk factors on kidney health can be detected and monitored by serial measurements of urine biomarkers. For instance, the associations of hemoglobin A1c with nearly all dimensions of tubular markers including tubular injury (higher IL-18), dysfunction (higher α1m), reserve (lower UMOD) and fibrosis (higher YKL-40), suggest the possibility of monitoring the various ways that hyperglycemia damages the kidney by measuring changes in these biomarker levels over time.

Findings from this analysis are in line with our prior cross-sectional study [26], and extend our earlier observations to longitudinal analyses, which allow for much stronger inferences. For instance, serum albumin is strongly predictive of mortality among PLWH [40–42], and the importance of serum albumin in our study was reflected by the association of both baseline and changes in serum albumin with biomarkers of tubular reabsorptive dysfunction, immune response and tubular reserve. To our knowledge, our current study is the first to report the associations of CKD risk factors with longitudinal changes in a comprehensive panel of urine biomarkers that capture various aspects of kidney health. One prior study reported on predictors of urine biomarker changes among women living with HIV, but only evaluated 3 urine biomarkers (IL-18, albumin-to-creatinine ratio and α1m) [43].

Urine biomarkers offer a path to a comprehensive assessment of kidney health beyond the current standard markers of kidney disease, eGFR and albuminuria, which generally reflect glomerular dysfunction and injury, respectively [1, 2]. Due to single-nephron compensatory hyperfiltration, reduction in eGFR is often only detected after significant kidney damage has occurred [44–46], and it is therefore limited for detection of early kidney disease [47–49]. In this study, changes in various biomarkers levels demonstrated evidence of kidney damage, even though only 2.6% of our study participants had eGFR < 60 ml/min at follow-up, indicating that these urine biomarkers detect kidney disease earlier than eGFR. Likewise, longitudinal increases in Ualb appeared to be influenced by systolic blood pressure and HIV viral load in this study. However, Ualb did not capture the effects of various other CKD risk factors that were reflected by changes in other biomarkers. Therefore, non-albumin urine biomarkers can complement albuminuria and eGFR in detecting and monitoring the effects of various CKD risk factors that are encountered in the care of HIV patients. Detection of subclinical injury can identify patients who may benefit from therapies to slow disease progression and avert associated adverse outcomes.

Our results should be interpreted in the context of our study’s limitations. First, we did not have kidney biopsies—the gold standard for diagnosing kidney disease [50]—and thus could not confirm the presence of kidney injury histologically. However, we focused on urinary biomarkers that have been associated with clinically relevant outcomes in persons living with HIV [12–21]. Second, we only had two serial urine biomarker measurements so assessment of longitudinal biomarker trajectories are limited. Third, our study may not have accounted for all potential risk factors and confounders, including exposure to other nephrotoxic medications. Fourth, this study is observational, so we cannot determine whether risk factor associations were causally related to biomarker changes. Lastly, we only included women living with HIV in this analysis, and thus our findings may not be generalizable to men living with HIV or individuals without HIV.

In conclusion, we have demonstrated that CKD risk factors are associated with distinct patterns of longitudinal changes in biomarker levels among women living with HIV. The contribution of these findings serves as an important step toward a biomarker guided strategy for monitoring kidney health in the setting of HIV infection. Future work should aim to identify the most parsimonious panel of biomarkers that can integrate longitudinal changes in biomarkers levels with clinical data to better detect and manage kidney disease risk within each individual.

## Supplementary Information

**Additional file 1: Supplemental Table 1.** Simultaneous multivariable adjusted associations of baseline and follow-up CKD risk factors with longitudinal changes in urine biomarker levels among HIV-positive women.

## Data Availability

The datasets used and/or analyzed during the current study are available from the corresponding author on reasonable request.

## References

[1] Levey AS, Eckardt KU, Tsukamoto Y, Levin A, Coresh J, Rossert J, Zeeuw DDE, Hostetter TH, Lameire N, Eknoyan G (2005). Definition and classification of chronic kidney disease: a position statement from kidney disease: improving global outcomes (KDIGO). Kidney Int.

[2] Rule AD, Amer H, Cornell LD, Taler SJ, Cosio FG, Kremers WK, Textor SC, Stegall MD (2010). The association between age and nephrosclerosis on renal biopsy among healthy adults. Ann Intern Med.

[3] Waikar SS, Betensky RA, Emerson SC, Bonventre JV (2012). Imperfect gold standards for kidney injury biomarker evaluation. J Am Soc Nephrol.

[4] Curthoys NP, Moe OW (2014). Proximal tubule function and response to acidosis. Clin J Am Soc Nephrol.

[5] Dantzler WH, Layton AT, Layton HE, Pannabecker TL (2014). Urine-concentrating mechanism in the inner medulla: function of the thin limbs of the loops of Henle. Clin J Am Soc Nephrol.

[6] Mount DB (2014). Thick ascending limb of the loop of Henle. Clin J Am Soc Nephrol.

[7] Subramanya AR, Ellison DH (2014). Distal convoluted tubule. Clin J Am Soc Nephrol.

[8] Pearce D, Soundararajan R, Trimpert C, Kashlan OB, Deen PM, Kohan DE (2015). Collecting duct principal cell transport processes and their regulation. Clin J Am Soc Nephrol.

[9] Roy A, Al-bataineh MM, Pastor-Soler NM (2015). Collecting duct intercalated cell function and regulation. Clin J Am Soc Nephrol.

[10] Bonventre JV, Vaidya VS, Schmouder R, Feig P, Dieterle F (2010). Next-generation biomarkers for detecting kidney toxicity. Nat Biotechnol.

[11] Waikar SS, Bonventre JV (2008). Biomarkers for the diagnosis of acute kidney injury. Nephron Clin Pract.

[12] Jotwani VK, Lee AK, Estrella MM, Katz R, Garimella PS, Malhotra R, Rifkin DE, Ambrosius W, Freedman BI, Cheung AK, Raphael KL, Drawz P, Neyra JA, Oparil S, Punzi H, Shlipak MG, Ix JH, for the SPRINT Research Group (2019). Urinary biomarkers of tubular damage are associated with mortality but not cardiovascular risk among systolic blood pressure intervention trial participants with chronic kidney disease. Am J Nephrol.

[13] Jotwani V, Scherzer R, Abraham A, Estrella MM, Bennett M, Cohen MH, Nowicki M, Sharma A, Young M, Tien PC, Ix JH, Sarnak MJ, Parikh CR, Shlipak MG (2015). Association of urine alpha1-microglobulin with kidney function decline and mortality in HIV-infected women. Clin J Am Soc Nephrol.

[14] Ascher SB, Scherzer R, Estrella MM, Zhang WR, Muiru AN, Jotwani V, Grunfeld C, Parikh CR, Gustafson D, Young M, Sharma A, Cohen MH, Ng DK, Palella FJ, Witt MD, Ho K, Shlipak MG (2018). Association of Urinary Biomarkers of kidney injury with estimated GFR decline in HIV-infected individuals following Tenofovir Disoproxil fumarate initiation. Clin J Am Soc Nephrol.

[15] Zhang WR, Scherzer R, Estrella MM, Ascher SB, Muiru A, Jotwani V, Grunfeld C, Parikh CR, Gustafson D, Kassaye S, et al. Tenofovir disoproxil fumarate initiation and changes in urinary biomarker concentrations among HIV-infected men and women. AIDS. 2019;33(4):723-33. 10.1097/QAD.0000000000002114. 10.1097/QAD.0000000000002114 PMC6400312 30830887

[16] Ascher SB, Scherzer R, Estrella MM, Shigenaga J, Spaulding KA, Glidden DV, Mehrotra ML, Defechereux P, Gandhi M, Grant RM, et al. HIV preexposure prophylaxis with tenofovir disoproxil fumarate/emtricitabine and changes in kidney function and tubular health. Aids. 2020;34(5):699-706. 10.1097/QAD.0000000000002456. 10.1097/QAD.0000000000002456 PMC7071971 31794523

[17] Garimella PS, Lee AK, Ambrosius WT, Bhatt U, Cheung AK, Chonchol M, Craven T, Hawfield AT, Jotwani V, Killeen A, Punzi H, Sarnak MJ, Wall BM, Ix JH, Shlipak MG (2019). Markers of kidney tubule function and risk of cardiovascular disease events and mortality in the SPRINT trial. Eur Heart J.

[18] Lee AK, Katz R, Jotwani V, Garimella PS, Ambrosius WT, Cheung AK, Gren LH, Neyra JA, Punzi H, Raphael KL, et al. Distinct Dimensions of Kidney Health and Risk of Cardiovascular Disease, Heart Failure, and Mortality. Hypertension. 2019;74(4):872-9. 10.1161/HYPERTENSIONAHA.119.13339. 10.1161/HYPERTENSIONAHA.119.13339 PMC6739187 31378102

[19] Malhotra R, Katz R, Jotwani V, Ambrosius WT, Raphael KL, Haley W, Rastogi A, Cheung AK, Freedman BI, Punzi H, Rocco MV, Ix JH, Shlipak MG (2020). Urine markers of kidney tubule cell injury and kidney function decline in SPRINT trial participants with CKD. Clin J Am Soc Nephrol.

[20] Ascher SB, Scherzer R, Estrella MM, Shlipak MG, Ng DK, Palella FJ, Witt MD, Ho K, Bennett MR, Parikh CR, Ix JH, Jotwani V (2019). Associations of urine biomarkers with kidney function decline in HIV-infected and uninfected men. Am J Nephrol.

[21] Jotwani V, Katz R, Ix JH, Gutiérrez OM, Bennett M, Parikh CR, Cummings SR, Sarnak MJ, Shlipak MG (2018). Urinary biomarkers of kidney tubular damage and risk of cardiovascular disease and mortality in elders. Am J Kidney Dis.

[22] Abraham AG, Althoff KN, Jing Y, Estrella MM, Kitahata MM, Wester CW, Bosch RJ, Crane H, Eron J, Gill MJ, Horberg MA, Justice AC, Klein M, Mayor AM, Moore RD, Palella FJ, Parikh CR, Silverberg MJ, Golub ET, Jacobson LP, Napravnik S, Lucas GM, North American AIDS Cohort Collaboration, Kirk GD, Benson CA, Bosch RJ, Collier AC, Boswell S, Grasso C, Mayer K, Hogg RS, Harrigan R, Montaner J, Cescon A, Brooks JT, Buchacz K, Gebo KA, Moore RD, Moore RD, Carey JT, Rodriguez B, Horberg MA, Silverberg MJ, Thorne JE, Goedert JJ, Jacobson LP, Klein MB, Rourke SB, Burchell A, Rachlis AR, Hunter-Mellado RF, Mayor AM, Gill MJ, Deeks SG, Martin JN, Saag MS, Mugavero MJ, Willig J, Eron JJ, Napravnik S, Kitahata MM, Crane HM, Justice AC, Dubrow R, Fiellin D, Sterling TR, Haas D, Bebawy S, Turner M, Gange SJ, Anastos K, Moore RD, Saag MS, Gange SJ, Althoff KN, Kitahata MM, McKaig RG, Justice AC, Freeman AM, Moore RD, Freeman AM, Lent C, Kitahata MM, van Rompaey SE, Crane HM, Webster E, Morton L, Simon B, Gange SJ, Althoff KN, Abraham AG, Lau B, Zhang J, Jing J, Golub E, Modur S, Hanna DB, Rebeiro P, Wong C, Mendes A, North American AIDS Cohort Collaboration (2015). End-stage renal disease among HIV-infected adults in North America. Clin Infect Dis.

[23] Estrella M, Fine DM, Gallant JE, Rahman MH, Nagajothi N, Racusen LC, Scheel PJ, Atta MG (2006). HIV type 1 RNA level as a clinical indicator of renal pathology in HIV-infected patients. Clin Infect Dis.

[24] Lucas GM, Jing Y, Sulkowski M, Abraham AG, Estrella MM, Atta MG, Fine DM, Klein MB, Silverberg MJ, Gill MJ, Moore RD, Gebo KA, Sterling TR, Butt AA, Kirk GD, Benson CA, Bosch RJ, Collier AC, Boswell S, Grasso C, Mayer K, Hogg RS, Harrigan R, Montaner J, Cescon A, Brooks JT, Buchacz K, Gebo KA, Moore RD, Carey JT, Rodriguez B, Horberg MA, Silverberg MJ, Horberg MA, Thorne JE, Goedert JJ, Jacobson LP, Klein MB, Rourke SB, Burchell A, Rachlis AR, Rico P, Hunter-Mellado RF, Mayor AM, Gill MJ, Deeks SG, Martin JN, Patel P, Brooks JT, Saag MS, Mugavero MJ, Willig J, Eron JJ, Napravnik S, Kitahata MM, Crane HM, Justice AC, Dubrow R, Fiellin D, Sterling TR, Haas D, Bebawy S, Turner M, Gange SJ, Anastos K, Moore RD, Saag MS, Gange SJ, Kitahata MM, McKaig RG, Justice AC, Freeman AM, Moore RD, Freeman AM, Lent C, Kitahata MM, van Rompaey SE, Crane HM, Webster E, Morton L, Simon B, Gange SJ, Althoff KN, Abraham AG, Lau B, Zhang J, Jing J, Golub E, Modur S, Hanna DB, Rebeiro P, Wong C, Mendes A, for the NA-ACCORD of the IeDEA (2013). Hepatitis C viremia and the risk of chronic kidney disease in HIV-infected individuals. J Infect Dis.

[25] Scherzer R, Estrella M, Li Y, Choi AI, Deeks SG, Grunfeld C, Shlipak MG (2012). Association of tenofovir exposure with kidney disease risk in HIV infection. AIDS..

[26] Muiru AN, Shlipak MG, Scherzer R, Zhang WR, Ascher SB, Jotwani V, Grunfeld C, Parikh CR, Ng D, Palella FJ, Ho K, Kassaye S, Sharma A, Cohen M, Wang R, Qi Q, Estrella MM (2019). Kidney disease risk factors associate with urine biomarkers concentrations in HIV-positive persons; a cross-sectional study. BMC Nephrol.

[27] Jotwani V, Scherzer R, Estrella MM, Jacobson LP, Witt MD, Palella FJ, Macatangay B, Bennett M, Parikh CR, Ix JH, Shlipak MG (2016). HIV infection, Tenofovir, and urine alpha1-microglobulin: a cross-sectional analysis in the multicenter AIDS cohort study. Am J Kidney Dis.

[28] Jotwani V, Scherzer R, Glidden DV, Mehrotra M, Defechereux P, Liu A, Gandhi M, Bennett M, Coca SG, Parikh CR, Grant RM, Shlipak MG (2018). Pre-exposure prophylaxis with Tenofovir Disoproxil fumarate/Emtricitabine and kidney tubular dysfunction in HIV-uninfected individuals. J Acquir Immune Defic Syndr.

[29] Ascher SB, Scherzer R, Nishtala A, Jotwani V, Grunfeld C, Parikh CR, Ng D, Wang R, Palella FJ, Shlipak MG, Estrella MM (2019). Association of Statin use with Kidney Damage and Function among HIV-infected men. J Acquir Immune Defic Syndr.

[30] Jotwani V, Scherzer R, Estrella MM, Jacobson LP, Witt MD, Palella F, Ho K, Bennett M, Parikh CR, Ix JH, Shlipak M (2017). Association of HIV infection with biomarkers of kidney injury and fibrosis in the multicenter AIDS cohort study. Antivir Ther.

[31] Barkan SE, Melnick SL, Preston-Martin S (1998). The Women's interagency HIV study. WIHS Collaborative Study Group. Epidemiology.

[32] Adimora AA, Ramirez C, Benning L (2018). Cohort Profile: The Women's Interagency HIV Study (WIHS). Int J Epidemiol.

[33] Obermüller N, Geiger H, Weipert C, Urbschat A (2014). Current developments in early diagnosis of acute kidney injury. Int Urol Nephrol.

[34] Vanmassenhove J, Vanholder R, Nagler E, Van Biesen W (2013). Urinary and serum biomarkers for the diagnosis of acute kidney injury: an in-depth review of the literature. Nephrol Dial Transplant.

[35] Lorenzen J, Shah R, Biser A, Staicu SA, Niranjan T, Garcia AM, Gruenwald A, Thomas DB, Shatat IF, Supe K, Woroniecki RP, Susztak K (2008). The role of osteopontin in the development of albuminuria. J Am Soc Nephrol.

[36] American DA (2020). 2. Classification and diagnosis of diabetes: standards of medical Care in Diabetes-2020. Diabetes Care.

[37] James PA, Oparil S, Carter BL, Cushman WC, Dennison-Himmelfarb C, Handler J, Lackland DT, LeFevre ML, MacKenzie TD, Ogedegbe O, Smith SC, Svetkey LP, Taler SJ, Townsend RR, Wright JT, Narva AS, Ortiz E (2014). 2014 evidence-based guideline for the management of high blood pressure in adults: report from the panel members appointed to the eighth joint National Committee (JNC 8). JAMA..

[38] Levey AS, Stevens LA, Schmid CH, Zhang Y(L), Castro AF, Feldman HI, Kusek JW, Eggers P, van Lente F, Greene T, Coresh J, for the CKD-EPI (Chronic Kidney Disease Epidemiology Collaboration) (2009). A new equation to estimate glomerular filtration rate. Ann Intern Med.

[39] Li Y, Nan B, Zhu J (2015). Multivariate sparse group lasso for the multivariate multiple linear regression with an arbitrary group structure. Biometrics..

[40] Lang J, Scherzer R, Weekley CC, Tien PC, Grunfeld C, Shlipak MG (2013). Serum albumin and short-term risk for mortality and cardiovascular disease among HIV-infected veterans. Aids..

[41] Rodriguez RA, Mendelson M, O'Hare AM, Hsu LC, Schoenfeld P (2003). Determinants of survival among HIV-infected chronic dialysis patients. J Am Soc Nephrol.

[42] Feldman JG, Gange SJ, Bacchetti P, Cohen M, Young M, Squires KE, Williams C, Goldwasser P, Anastos K (2003). Serum albumin is a powerful predictor of survival among HIV-1-infected women. J Acquir Immune Defic Syndr.

[43] Baxi SM, Scherzer R, Jotwani V, Estrella MM, Abraham AG, Parikh CR, Bennett MR, Cohen MH, Nowicki MJ, Gustafson DR, Sharma A, Young MA, Shlipak MG, for the Women's Interagency HIV Study (WIHS) (2017). Changes in urinary biomarkers over 10 years is associated with viral suppression in a prospective cohort of women living with HIV. J Acquir Immune Defic Syndr.

[44] Helal I, Fick-Brosnahan GM, Reed-Gitomer B, Schrier RW (2012). Glomerular hyperfiltration: definitions, mechanisms and clinical implications. Nat Rev Nephrol.

[45] Melsom T, Stefansson V, Schei J, Solbu M, Jenssen T, Wilsgaard T, Eriksen BO (2016). Association of Increasing GFR with change in albuminuria in the general population. Clin J Am Soc Nephrol.

[46] Denic A, Mathew J, Lerman LO, Lieske JC, Larson JJ, Alexander MP, Poggio E, Glassock RJ, Rule AD (2017). Single-nephron glomerular filtration rate in healthy adults. N Engl J Med.

[47] Steffl JL, Bennett W, Olyaei AJ (2012). The old and new methods of assessing kidney function. J Clin Pharmacol.

[48] Cirillo M (2010). Evaluation of glomerular filtration rate and of albuminuria/proteinuria. J Nephrol.

[49] Bellomo R, Kellum JA, Ronco C (2004). Defining acute renal failure: physiological principles. Intensive Care Med.

[50] Luciano RL, Moeckel GW (2019). Update on the native kidney biopsy: Core curriculum 2019. Am J Kidney Dis.

