# Supplementary material for: Associations of CKD risk factors and longitudinal changes in urine biomarkers of kidney tubules among women living with HIV
Source: BMC Nephrol. 2021 Aug 30;22:296. doi: 10.1186/s12882-021-02508-6 (PMC8406753; doi:10.1186/s12882-021-02508-6)
Supplement: Supplementary file 1 — Additional file 1: Supplemental Table 1. Simultaneous multivariable adjusted associations of baseline and follow-up CKD risk factors with longitudinal changes in urine biomarker levels among HIV-positive women. [file 12882_2021_2508_MOESM1_ESM.docx]

| **Supplemental Table 1: Simultaneous multivariable adjusted associations of baseline and follow-up CKD risk factors with longitudinal changes in urine biomarker levels among HIV-positive women** | | | | | | | | | | | | | | |
| --- | --- | --- | --- | --- | --- | --- | --- | --- | --- | --- | --- | --- | --- | --- |
| **Urine Biomarkers** | | | | | | | | | | | | | | |
|  | U Alb | OPN | α1m | B2m | UcysC | TFF3 | KIM1 | IL-18 | Clusterin | NGAL | UMOD | EGF | MCP-1 | YKL-40 |
| **CKD risk factors** | **Estimate**  **(95%CI)** | | | | | | | | | | | | | |
| Age |  |  | 0.05  (0.01, 0.10) |  |  |  |  |  |  |  |  |  | 0.10  (0.05, 0.15) |  |
| Black race |  | 0.15  (0.04, 0.25) |  |  |  |  |  |  |  |  |  |  |  |  |
| Diabetes |  |  |  |  |  |  |  |  |  |  |  | -0.18  (-0.29, -0.06) |  |  |
| a1c |  |  | 0.06  (0.01, 0.11) |  |  |  |  | 0.06  (0.02, 0.11) |  | 0.07  (0.004, 0.13) | -0.10  (-0.16, -0.03) |  |  | 0.10  (0.04, 0.17) |
| Change in a1c |  |  |  |  |  |  | 0.04  (0.002, 0.08) | 0.05  (0.01, 0.10) |  | 0.09  (0.02, 0.15) |  |  | 0.07  (0.02, 0.12) | 0.13  (0.06, 0.19) |
| HTN | 0.15  (0.03, 0.27) |  |  |  |  |  |  |  |  |  |  |  |  |  |
| Systolic BP | 0.06  (0.01, 0.12) |  |  |  |  |  |  |  |  |  |  | -0.08  (-0.13, -0.03) |  |  |
| Change in systolic BP |  |  |  |  | -0.07  (-0.11, -0.03) |  |  |  |  |  |  |  |  |  |
| Diastolic BP |  |  |  |  |  |  | 0.05  (0.01, 0.10) |  |  |  |  |  |  |  |
| Change in diastolic BP |  |  |  |  |  |  | -0.05  (-0.10, -0.01) |  |  | -0.09  (-0.14, -0.03) | -0.08  (-0.15, -0.02) |  |  |  |
| HDL |  |  |  |  |  |  | -0.07  (-0.11, -0.03) |  |  |  |  |  |  |  |
| Change in HDL |  |  |  |  |  |  |  | -0.06  (-0.10, -0.02) |  |  |  |  |  |  |
| LDL |  |  |  | -0.05  (-0.10, -0.01) |  |  |  |  |  |  |  |  |  |  |
| BMI |  | -0.07  (-0.11, -0.01) | -0.06  (-0.11, -0.01) |  |  |  |  | 0.05  (0.01, 0.10) |  |  |  |  |  |  |
| Change in BMI | 0.07  (0.01, 0.12) | 0.07  (0.02, 0.11) |  |  |  |  |  |  |  |  |  |  |  |  |
| Serum albumin |  |  |  |  | -0.13  (-0.18, -0.08) |  | -0.08  (-0.13, -0.03) |  |  | -0.12  (-0.19, -0.05) |  | 0.09  (0.04, 0.14) | -0.10  (-0.15, -0.04) | -0.17  (-0.25, -0.10) |
| Change in serum albumin |  |  | -0.10  (-0.16, -0.04) | -0.08  (-0.14, -0.02) |  | -0.18  (-0.26, -0.11) |  |  | -0.06  (-0.10, -0.01) |  | 0.13  (0.05, 0.20) |  |  |  |
| HCV |  |  |  | 0.31  (0.19, 0.43) |  | 0.21  (0.06, 0.36) |  |  |  |  |  |  |  | 0.23  (0.08, 0.37) |
| CD4 |  |  |  | -0.08  (-0.12, -0.03) |  |  |  | -0.09  (-0.13, -0.05) |  |  | 0.09  (0.02, 0.16) |  |  |  |
| Change in CD4 |  |  | -0.09  (-0.14, -0.03) |  | -0.05  (-0.10, -0.01) |  |  |  |  |  | -0.08  (-0.15, -0.001) |  | -0.07  (-0.12, -0.02) |  |
| HIV RNA | 0.06  (0.01, 0.11) |  |  |  |  |  | 0.05  (0.003, 0.09) |  |  |  |  |  |  |  |
| Change in HIV RNA |  |  |  | 0.11  (0.07, 0.16) |  |  |  | 0.18  (0.14, 0.22) |  |  |  |  |  |  |
| HIV Duration |  |  |  |  |  |  | 0.09  (0.05, 0.13) |  |  |  |  |  |  |  |
| TDF duration |  |  |  | 0.11  (0.05, 0.16) |  | 0.09  (0.03, 0.15) |  |  |  |  |  |  |  | -0.07  (-0.13, -0.02) |
| ART duration |  |  |  | -0.08  (-0.13, -0.02) |  |  |  |  |  |  |  |  |  |  |
| Ritonavir duration |  |  |  |  |  |  |  |  |  |  | 0.09  (0.02, 0.15) |  |  |  |

We modeled biomarkers in combination using the multivariable sparse group least absolute shrinkage and selection operator (MSG-LASSO) method for variable selection. Blank boxes indicate variables that were not selected by the MSG-LASSO method. Estimates are reported as standardized regression coefficients (e.g 1 standard deviation (SD) increase in hemoglobin a1c is associated with a 0.06 SD increase in α1m). a1c: hemoglobin a1c; HTN: hypertension; BP: Blood pressure; LDL: Low density lipoprotein; HDL: high density lipoprotein; BMI: Body mass index; HCV: hepatitis C virus; TDF: tenofovir disoproxil fumarate; ART: Anti-retroviral therapy; UAlb: Urine albumin; OPN: osteopontin; α1m: α1-microglobulin; β2m: β2-microglobulin; UcysC: Urine cystatin C; TFF3: Trefoil factor 3; KIM-1: kidney injury marker-1; IL-18: interleukin 18; NGAL: Neutrophil gelatinase-associated lipocalin; UMOD: uromodulin; EGF: epidermal growth factor; MCP1: Monocyte chemoattractant protein-1; YKL-40: chitinase-3-like protein-1
